# Lung Transplantation for Pleuroparenchymal Fibroelastosis: A Single-Center Experience with Revision of Literature

**DOI:** 10.3390/biomedicines11061505

**Published:** 2023-05-23

**Authors:** Eleonora Faccioli, Vincenzo Verzeletti, Chiara Giraudo, Marco Schiavon, Fiorella Calabrese, Monica Loy, Federico Rea, Andrea Dell’Amore

**Affiliations:** 1 Thoracic Surgery and Lung Transplant Unit, Department of Cardiac-Thoracic-Vascular Sciences and Public Health, University Hospital of Padua, 35128 Padua, Italy; vincenzo.verzeletti@aopd.veneto.it (V.V.); marco.schiavon@unipd.it (M.S.); monica.loy@aopd.veneto.it (M.L.); federico.rea@unipd.it (F.R.); andrea.dellamore@unipd.it (A.D.); 2 Radiology Unit, Department of Medicine, University Hospital of Padua, 35128 Padua, Italy; chiara.giraudo@unipd.it; 3 Pathology Unit, Department of Cardiac-Thoracic-Vascular Sciences and Public Health, University Hospital of Padua, 35128 Padua, Italy; fiorella.calabrese@unipd.it

**Keywords:** lung transplantation, pleuroparenchymal fibroelastosis, outcomes

## Abstract

Pleuroparenchymal fibroelastosis (PPFE) is a rare condition characterized by fibrosis involving the pleura and the upper lobes which can be idiopathic or secondary to chemotherapy, transplantations and occupational exposure. For the end-stage form, lung transplantation (LT) is the treatment of choice. The aim of this study was to report our single-center experience for patients subjected to LT for PPFE and comparing it with the already published evidence on this topic. At our center, we have performed 6 bilateral LTs for patients with PPFE (3 males and 3 females) with a median age of 52 years. Median ICU and in-hospital length of stay were 8 and 30 days, respectively. To date, two patients are alive and four are dead, with a median overall survival of 10 months. In addition, after a formal search using the terms “pleuroparenchymal fibroelastosis AND lung transplantation”, we collected 14 studies focused on outcomes after LT. LT for PPFE is technically challenging and its post-operative course could also be complicated. Current available data on LT outcomes are extremely poor and mostly limited to case reports. Further studies need to be published to improve knowledge of this disease and to achieve best outcomes for LT.

## 1. Introduction

Pleuroparenchymal fibroelastosis (PPFE) is a pulmonary disease that was formally classified as a rare idiopathic interstitial pneumonia in 2013 [1]. This clinical entity is characterized by fibrotic changes in the lung associated with pleural thickening, typically involving the upper lobes with sparing of the parenchyma distant from the pleura. The clinical features of the disease are peculiar; affected patients are thin, with low body mass index and a typical chest wall configuration known as “flat chest” [2], defined as an abnormally lower ratio of the anteroposterior to the transverse diameter of the thoracic cage which tends to worsen during the progression of the disease but which can be reversed after lung transplantation [3]. PPFE can be idiopathic or as a consequence of bone marrow and hematopoietic cell transplantations [4] in association with chemotherapy drugs [5,6], occupational exposure [7] or recurrent pulmonary infections [8]. In some cases, especially in young women, it can also occur in the context of familial forms of idiopathic interstitial pneumonia [9].

Regardless of the etiology, the clinical course is often severe and progressive with a restrictive impairment: the first symptoms are usually cough and dyspnea, while recurrent pneumothoraces can occur in advanced stages of the disease with the presence of multiple bullae and large cysts. This disease is usually refractory to steroids and other medical treatments, so lung transplantation (LT) is considered the treatment of choice for patients with end-stage respiratory failure related to this disease. Technical aspects (such as adhesions and chest wall rigidity) and peculiar clinical features of PPFE such as chronic and recurrent pulmonary infections, especially those due to Aspergillus and nontuberculous mycobacteria, low body mass index, and associated autoimmune or connective tissue diseases can make the transplantation challenging with poor post-LT outcomes [10,11,12]; for these reasons, very little data, especially case reports, are available regarding the outcomes of LT in PPFE patients.

The aim of our study was to report our single-center experience with LT in patients affected by PPFE, with a focus on short- and long-term outcomes. In addition, we have performed a review including all the studies currently available concerning outcomes of LT for PPFE. Since data published so far on this topic are scarce and heterogeneous, this study could improve knowledge of and evidence for LT for this rare indication.

## 2. Materials and Methods

### 2.1. Patients and Methods

This study was approved by the Institutional Review Board (IRB) of our center (4539/AO/18). The need for informed consent was waived because of the retrospective nature of this study. All the six patients enrolled in this case series were adults affected by pathologically proven PPFE (idiopathic or as late onset complication of hematopoietic stem cell transplantation) on the native lung who underwent bilateral lung transplantation at the Thoracic Surgery Unit of the University Hospital of Padua, Italy. Complete clinical and radiological information was available for all patients. From a radiological point of view, the patients were assessed according to the criteria proposed by Reddy et al. [8], while from the pathological point of view, the diagnostic criteria were (1) intense fibrosis of the visceral pleura, (2) prominent, homogeneous, subpleural fibroelastosis, (3) sparing of the parenchyma distant from the pleura, (4) mild patchy lymphoplasmacytic infiltrates and (5) small numbers of fibroblastic foci present at the leading edge of the fibrosis [9].

Clinical pre-, intra- and post-operative data were collected from medical records for each patient and recorded in a Microsoft Excel database. 3D reconstructions of the patients’ chest walls were obtained after a semi-automatic segmentation process using Materialise Mimics InPrint (Materialise NV, Leuven, Belgium) to see if chest-wall flatness was reversed after LT. All the patients’ data are reported in Table 1 and Table 2.

### 2.2. Review of Current Literature

A review of the literature was conducted using a formal strategy (https://www.ncbi.nlm.nih.gov/pubmed; accessed on 30 December 2022).

To retrieve all the publications dealing with the topic of interest, the query string was composed as “Pleuroparenchymal Fibroelastosis AND Lung Transplantation”. The aim of our research was to report short- and long-term outcomes of patients affected by PPFE and submitted to lung transplantations; however, since few studies were focused on this aspect, we could not perform a proper systematic review according to the Reporting Items for Systematic Reviews and Meta-Analysis (PRISMA) statement [13].

All the eligible studies were focused on transplanted patients and concerned pre-operative assessment, intra-operative course and post-operative outcomes. After the search using the keywords mentioned above, a total of 14 studies were identified, most of which were single-case reports, with only one study reporting an international experience with 31 patients. All the available studies and outcomes are reported in Table 3.

## 3. Results

### 3.1. Case Presentations

At the Thoracic Surgery Unit and Lung Transplant Center of the University Hospital of Padua, six patients affected by PPFE were submitted to LT. The detailed description of each case is reported below.

#### 3.1.1. Patient 1

The first patient is a 62-year-old male who was listed for LT in March 2021 at our center for pleuroparenchymal pulmonary fibroelastosis. He was a former smoker, and his past medical history was characterized by occupational exposure to hydrocarbon dust and vapors; in addition, in 2012, the patient underwent a right lower lobectomy for a mucinous lung adenocarcinoma (pT2bN0) without any adjuvant therapy. After a waiting list time of 469 days, he successfully underwent bilateral LT with a lung allocation score (LAS) of 32.9. The transplant was led with the prophylactic assistance of intra-operative central veno-arterial extracorporeal membrane oxygenation (ECMO), which was removed at the end of the surgical procedure. The transplantation was complicated by the presence of tenacious bilateral adhesions, which resulted in major blood loss and the consequent need for copious blood transfusions.

The definitive histological examination of the native lungs confirmed the previous diagnosis of idiopathic pleuroparenchymal fibroelastosis. The postoperative course was characterized by the need for prolonged mechanical ventilation for which a tracheostomy was performed on post-operative day (POD) 10. The intensive care unit (ICU) and in-hospital length of stay were 12 and 32 days, respectively. To date, the patient is alive, in good general condition with a forced vital capacity (FVC) of 60% and no signs of rejection.

#### 3.1.2. Patient 2

The second patient is a non-smoking 61-year-old female with an idiopathic PPFE, for which she was in continuous oxygen therapy since January 2021. In January 2022, she underwent a bilateral LT with an LAS of 33.9 after a waiting list time of 144 days. The transplantation was conducted within a surgical time of 320 min and without intra-operative complications with prophylactic central ECMO support, which was removed at the end of the procedure.

The patient was extubated on POD 1, discharged from the ICU on POD 4 and from the hospital on POD 28. At the last follow-up, 9 months after the LT, respiratory function was satisfactory, with a FVC of 87% of the predicted value and no signs of chronic lung allograft dysfunction (CLAD). However, the patient died 393 days after the LT due to multi-organ failure consequent to sepsis.

#### 3.1.3. Patient 3

The third patient is a 51-year-old non-smoking female affected by idiopathic PPFE without any other comorbidity but with a high need for continuous oxygen therapy.

After a waiting list time of 456 days and with an LAS of 38.55, she underwent a bilateral lung transplant in September 2021.

The surgical procedure lasted 350 min and was led with prophylactic intraoperative central ECMO assistance.

**Figure 1:**
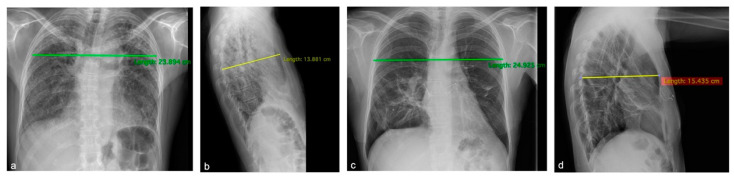
Patient Nº3. Chest X-rays pre- (**a**,**b**) and 18 months post- (**c**,**d**) lung transplantation show the modifications of the thoracic cage diameters with a reversion of the flat chest.

**Figure 2:**
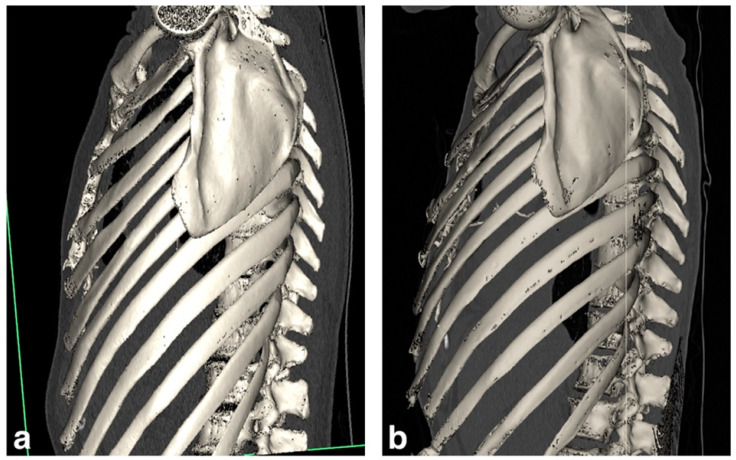
Patient Nº3. 3D reconstructions obtained from CT scans pre- (**a**) and 18 months post- (**b**) lung transplantation highlight the reversion of the flat chest of the patient.

The postoperative course was uneventful: the patient was extubated on POD 2, discharged from the ICU on POD 5 and from the hospital on POD 30. Currently, the patient is alive, with good respiratory function and with no signs of rejection at the last follow-up almost 3 years after the LT. In addition, this patient before transplantation had a significantly flattened chest cage which improved after LT, as shown in Figure 1 and Figure 2.

#### 3.1.4. Patient 4

The fourth patient is a man of 53 years with terminal respiratory failure and a diagnosis of unusual interstitial pneumonia (UIP) made in 2018 from transbronchial biopsies. After a waiting list time of 29 days and with an LAS of 36.9, he successfully underwent a double lung transplant without any intra-operative complications and with a surgical time of 345 min.

The final pathological examination of native lungs confirmed the initial diagnosis of UIP but also revealed diffused foci of pleuroparenchymal fibroelastosis. The patient was extubated on POD 1 and discharged from the ICU on POD 4. However, on POD 9 the patient was re-operated for hemothorax. He was discharged on POD 31 in good general condition with adequate pulmonary function but, unfortunately, he died from COVID-19 pneumonia six months after transplantation.

#### 3.1.5. Patient 5

The fifth patient is a 51-year-old male non-smoker affected by idiopathic PPFE. His past medical history was silent, except for a Ravitch procedure for pectus excavatum in 2008. In 2017, the patient received a double LT at another center without complications and with an initial good recovery of his respiratory function. Six months after the first LT, he developed CLAD, for which he was re-listed for a new double LT, performed in January 2018. Unfortunately, one year later, his clinical condition worsened again: a new diagnosis of bronchiolitis obliterans syndrome (BOS) was made for which the patient received steroid therapy and photopheresis with no results. The patient was then referred to our center for a third LT.

In February 2020, when the northern Italian regions were in the midst of the first COVID-19 wave, a matching donor was proposed to our center. The third LT was technically challenging because of diffuse adhesions due to the previous surgeries and lasted 560 min; a middle lobectomy and lingulectomy were also performed because of a size mismatch between the donor and the recipient (donor/recipient TLC ratio 2.05). The LT was conducted with the assistance of a central ECMO which was interrupted at the end of surgery.

The post-transplantation course was extremely complicated and characterized by hemothorax on POD 1 which required a surgical reintervention, the need for renal ultrafiltration, and the onset of atrial fibrillation, for which the patient underwent both pharmacological and electrical cardioversion. In addition, his respiratory functions continued to worsen and microbiological examination of his bronchoalveolar lavage showed the presence of fungal hyphae. The patient died from Aspergillus pneumonia on POD 28.

#### 3.1.6. Patient 6

The sixth patient is a 39-year-old non-smoking woman with a PPFE secondary to a previous bone marrow transplantation for acute lymphoblastic leukemia in 1997.

The patient was listed at our center for bilateral LT in June 2022. After 135 days on the waiting list, her respiratory condition suddenly deteriorated; thus, she was intubated and put on peripheral veno-venous ECMO assistance. The patient was re-listed on the national emergency waiting list and received a double lung transplant 11 days later.

The intra-operative course of LT, conducted with central VA ECMO, was extremely complicated, and the presence of diffuse adhesions at level of the left hilum caused massive bleeding with a subsequent transitory cardiac arrest. Once the patient was hemodynamically stabilized, the ECMO support was switched to a peripheral veno-arterial configuration.

The post-operative course was characterized by two episodes of hemothorax (on POD 2 and 10), both of which required surgical re-interventions, and the need for continuous veno-venous hemofiltration (CVVH). The peripheral veno-arterial ECMO was removed on POD 8 and replaced with a Protek Duo^®^ cannula (TandemLife, Pittsburg, PA, USA) because of residual right heart dysfunction. On POD 10, given the need for prolonged mechanical ventilation, the patient was tracheostomized. Unfortunately, the patient died from multi-organ failure (MOF) on POD 18.

### 3.2. Revision of the Available Literature

To the best of our knowledge, up to now, there have been 14 published studies with a total of 44 patients that are focused on outcomes after LT for PPFE. Among these, only one [22], with a total of 31 patients affected by PPFE and submitted for LT, is a multicentric study reporting a national experience; the other 13 studies are single-case reports. Twenty-two patients (50%) were female, and the median age at LT was 27.5 years (IQR 25.3–37.5).

Thirty-nine patients (89%) were affected by idiopathic PPFE, while five (11%) had a secondary form consequent to stem cell transplantation or chemotherapy administration.

The majority of patients (31; 70%) underwent bilateral LT, while in 13 cases (30%) a single LT was performed.

Seven studies reported the duration of in-hospital stay after LT, with a median value of 37.5 days (IQR 27.3–104), while twelve presented data concerning in-hospital mortality, which occurred only in one patient. Concerning long-term survival, at the time of publication of the abovementioned studies, 28 patients (64%) were alive and 13 (29%) were dead, while for 3 patients (7%), this datum was not reported. The median overall survival (OS) after LT was 24.5 months (IQR 9–24). All the available studies and outcomes are reported in Table 3.

## 4. Discussion

PPFE is an insidious disease, especially from both a diagnostic and clinical point of view. For this reason, the first important consideration is that the low number of patients subjected to LT for this pathology is certainly underestimated, and this explains why so far, we have only found a total of 44 published cases, which has now reached 50 with our case series of 6 patients. The main point that has emerged from our research is that the results after lung transplantation for PPFE are still heterogenous and some clinical aspects of this disease need to be further investigated.

To date, no international consensus regarding the diagnostic criteria of the disease have been established [22], and the real prevalence and incidence of the disease remains unknown. In our case series, we collected only six patients affected by PPFE and submitted for LT but other previous patients could certainly have been diagnosed with other forms of idiopathic interstitial pneumonia (IIP); for this reason, a retrospective pathological revision of all the interstitial diseases in the native explanted lungs would be desirable to determine the exact incidence of this entity [24,25].

PPFE is a pathology with a long subclinical stage [26], and the proper timing to refer a patient to a LT center is still unknown, even though, in most cases, the same criteria used for the referral in the case of IPF are utilized [27,28]. At the same time, post-operative outcomes after LT are not so homogeneous, especially because the reported experiences are limited to single-case reports and highly influenced by the different center’s expertise and techniques in lung transplantation. The studies published so far have reported 64% of patients still alive after LT, with a median overall survival of 24 months. In our case series, two patients died, and the OS is shorter (10 months), but we have to consider that the two early deaths occurred in patients who arrived for LT in extremely critical clinical conditions (one was mechanically ventilated and supported by VV ECMO and the other one was undergoing his third LT due to BOS). In addition, the median age of our transplanted patients was notably higher than those reported by the other 14 studies (56 vs 27.5 years old), and the first patient enrolled in our case series was transplanted in 2020, so the follow-up was unavoidably shorter. Again, one of the two patients with the worst outcome (patient Nº6) had a PPFE secondary to a bone marrow transplantation 25 years before the LT. Data on outcomes after LT for secondary PPFE are very poor: in the current literature, only five cases were transplanted for secondary PPFE [14,15,16,19,20] and among these, only four authors reported post-LT survival, with a median OS of 8 months. Zhang et al. [29] reported a case of a patient affected by secondary PPFE to autologous stem cell transplantation who succumbed to respiratory failure and infection while awaiting LT. A recent study [30] compared patients affected by idiopathic PPFE with those having secondary PPFE and, despite a higher incidence of usual interstitial pneumonia (UIP) in the lower lobes in those affected by secondary PPFE, the authors found no differences in terms of laboratory and respiratory data, complications and survival between the two groups. They thus concluded that LT should be proposed in young patients with secondary forms of PPFE with low DLCO, regardless of their lower-lobe ILD pattern. Few patients are affected by secondary forms, and the number of cases submitted for LT are even fewer, so strong conclusions cannot be drawn. Despite the scarcity of evidence, the general idea is that the prognosis of these patients remains very poor because of the diagnostic delays and the frequent acute exacerbations of the disease; for these reasons, it may be advisable to refer the patient as early as possible to LT center.

The other important point, which is also one of the main reported features in patients affected by PPFE, relates to a characteristic chest wall deformity known as flat chest [2]. This abnormality could worsen the progression of the disease, deteriorating the patient’s clinical conditions at the time of LT due to the shrinkage of upper lobes through fibrosis and probably increasing the negative intrathoracic pressure, making the surgical procedure more challenging. Shiiya et al. [23] recently described an extreme case of flat chest in a PPFE patient: this dynamic anatomical change caused a mediastinal shift with a consequent pulmonary hypertension due to the obstruction of the outflow tract of the pulmonary vein. The correction of this thoracic cage abnormality has already been described after lung transplantation [3]. In our case series, one patient (patient Nº5) was subjected to a Ravitch procedure nine years before his first LT because of this symptomatic chest wall deformity, and another patient (patient Nº3) had radiological and clinical evidence of flat chest which significantly improved after LT, as shown in Figure 1 and Figure 2. These two patients had the worst FVC before LT and this suggests that, in PPFE, lung volume itself may be worsened by the rigidity of the chest wall. Regarding post-LT respiratory function, despite us reporting an improvement in FVC at follow-up for every patient (median pre-LT, 44%; median post-LT, 86%), it has been described that this inability might persist and also limit the lung function after LT: an intensive pulmonary rehabilitation is generally recommended even if the chest wall flatness can be reversed after LT [3,18].

## 5. Conclusions

This study summarizes all the available evidence reported so far in the current literature regarding the outcomes of LT for PPFE. The number of reported cases is also very small considering that the exact prevalence of this disease is underestimated, since it is often labelled as generic IIP. Given the small numbers of patients subjected to LT, no definitive conclusions can be drawn on post-operative outcomes; however, these patients seem to have challenging intra and short-term results, although their long-term outcomes are essentially similar to other ILDs. LT could be considered in advanced stages both in idiopathic and secondary PPFE, and post-operative rehabilitation, especially for the peculiar conformation of the chest cavity, should always be performed. Even though further multicentric studies are needed to increase knowledge of this disease, this research could improve knowledge on this disease and help clinicians in the selection of the adequate LT candidate and the post-operative management of these complex patients.

## Figures and Tables

**Table 1:** Baseline characteristics of the patients transplanted in our center due to PPFE.

Patient	Age	Sex	BMI	LAS	Waiting List Time (Days)	Diagnosis	Time from Diagnosis to LT (Months)	FVC Pre (%)	DLCO Pre (%)	mPAP (mmHg)
# 1	62	M	19.8	32.9	469	IPPFE	72	47	38	14
# 2	61	F	18.2	33.9	144	IPPFE	84	58	23	13.5
# 3	51	F	19.8	38.5	456	IPPFE	60	31	20	20
# 4	53	M	21.2	36.9	29	UIP + IPPFE	12	56	24	10
# 5	51	M	18.5	41.1	90	BOS in IPPFE	12 *	41	46	17
# 6	39	F	19.5	50.8	136	Secondary PPFE	10	13	30	32

BMI: body mass index; BOS: bronchiolitis obliterans syndrome; DLCO: diffusion lung carbon monoxide; FVC: forced vital capacity; IPPFE: idiopathic pleuroparenchymal fibroelastosis; LAS: lung allocation score; mPAP: middle pulmonary artery pressure; PPFE: pleuroparenchymal fibroelastosis; UIP: unusual interstitial pneumonia; * the time indicated refers to the time elapsed between diagnosis and first transplantation.

**Table 2:** Intraoperative, postoperative and follow-up data of the patients transplanted in our center due to PPFE.

LT Type	LT Time (min)	ECMO			MV (h)	ICU Stay (Days)	In H Stay (Days)	* PGD	CLAD	** FVC Post (%)	** DLCO Post (%)	OS (Months)	Status	Death Cause
		Pre	Intra	Prolonged										
BLT	580	No	Yes	No	280	12	32	0	No	73	35	7	Alive	-
BLT	320	No	Yes	No	16	4	28	0	No	87	39	13	Death	MOF
BLT	350	No	Yes	No	40	5	30	0	No	86	44	29	Alive	-
BLT	345	No	Yes	No	18	4	31	0	No	86	44	6	Death	COVID-19 Pneumonia
BLT	560	No	Yes	No	560	38	38	3	-	-	-	0	Death	Aspergillus Pneumonia
BLT	410	Yes	Yes	Yes	480	18	18	3	-	-	-	0	Death	MOF

BLT: bilateral lung transplant; CLAD: chronic lung allograft dysfunction; DLCO: diffusion lung carbon monoxide; ECMO: extracorporeal membrane oxygenation; FVC: forced vital capacity; H: hospital; ICU: intensive care unit; LT: lung transplant; MOF: multi-organ failure; MV: mechanical ventilation; OS: overall survival; PGD: primary graft dysfunction. * at 72 h after LT; ** at last follow up.

**Table 3:** Case report/case series reporting outcomes after LT for PPFE.

Author, Year	Nº of Reported Cases	Age	Sex	Diagnosis	Time from Diagnosis to LT (Months)	Type of LT	In H Stay (Days)	In H Mortality (Days)	* Status	OS (Months)
Chen, 2014 [14]	1	14	M	Secondary PPFE	After LT	Left SLT	NR	No	Alive	4
Portillo, 2015 [15]	1	25	M	Secondary PPFE	14	BLT	NR	No	Alive	24
Yanagiya, 2016 [3]	1	27	F	IPPFE	3	LDLLT	NR	No	Alive	6
Hata, 2016 [16]	1	19	M	Secondary PPFE	60	LDLLT	25	No	Alive	1
Huang, 2017 [17]	1	36	M	IPPFE	36	BLT	NR	No	Alive	NR
Aljefri, 2018 [11]	1	27	M	IPPFE	After LT	BLT	45	No	NR	NR
Righi, 2018 [18]	1	42	M	IPPFE	36	BLT	30	No	Alive	24
Shimada, 2018 [19]	1	21	F	Secondary PPFE	24	LDLLT	120	No	Alive	12
Tsubosaka, 2018 [20]	1	19	M	Secondary PPFE	24	LDLLT	NR	NR	NR	NR
Ali, 2019 [10]	1	26	F	IPPFE	24	BLT	NR	No	Alive	44
Rasciti, 2019 [21]	1	48	M	IPPFE	12	First BLT Re-BLT	20 28	No No	Alive	12 (from Re.LT)
Sekine, 2020 [12]	1	29	F	IPPFE	36	LDLLT	365	No	Alive	24
Shiiya, 2021 [22]	31	51 **	F 58% M 42%	IPPFE	NR	65% BLT 35% SLT	99 **	3%	61% Alive 39% Dead	~100
Shiiya, 2022 [23]	1	40	F	IPPFE	NR	Left SLT	120	No	Dead	18

BLT: bilateral lung transplantation; IPPFE: idiopathic pleuroparenchymal fibroelastosis; LDLLT: living-donor lobar lung transplantation; LT: lung transplantation; NR: not reported; OS: overall survival; PPFE: pleuroparenchymal fibroelastosis; SLT: single lung transplantation; * at the time of publication; ** median value.

## Data Availability

The data reported in this study are unavailable due to privacy and ethical restrictions.
